# A Plug-and-Play Solution for Smart Transducers in Industrial Applications Based on IEEE 1451 and IEC 61499 Standards

**DOI:** 10.3390/s22197694

**Published:** 2022-10-10

**Authors:** Diogo Oliveira, João Pinheiro, Luís Neto, Vítor H. Pinto, Gil Gonçalves

**Affiliations:** 1 FEUP—Faculty of Engineering, University of Porto, Rua Dr. Roberto Frias, 4200-465 Porto, Portugal; 2 SYSTEC (DIGI2)—Research Center for Systems and Technologies (Digital and Intelligent Industry Lab), 4200-465 Porto, Portugal

**Keywords:** smart transducer, plug and play, IEEE 1451, industrial applications, IEC 61499

## Abstract

In a cyberphysical production system, the connectivity between the physical entities of a production system with the digital component that controls and monitors that system takes fundamental importance. This connectivity has been increasing from the transducers’ side, through gathering new functionalities and operating increasingly independently, taking the role of smart transducers, and from the applications’ side, by being developed in a distributed and decentralized paradigm. This work presents a plug-and-play solution capable of integrating smart transducers compliant with the IEEE 1451 standard in industrial applications based on the IEC 61499 standard. For this, we implemented the NCAP module of the smart transducer defined in IEEE 1451, which, when integrated with 4diac IDE and DINASORE (development and execution tools compliant with IEC 61499), enabled a solution that presented automatically the smart sensors and actuators in the IDE application and embedded their functionalities (access to data and processing functions) in the runtime environment. In this way, a complete plug-and-play solution was presented from the connection of the transducer to the network until its integration into the application.

## 1. Introduction

A cyberphysical production system (CPPS) is a manufacturing system, in which the physical system (sensors and actuators at the field level) is tightly integrated with the cyber system (e.g., ongoing processes, production management, and condition monitoring), decentralizing and distributing the computation entities among a mesh network of nodes and subsystems [1]. This allows taking advantage of data-accessing and data-processing services driven by the most recent developments in computer science, information and communication technologies, artificial intelligence, and machine learning [2].

In a decentralized and distributed system, the independence of the different nodes that compose the system can be applied down to the transducers’ level, typically composed of sensors and actuators that do not integrate the most advanced information and communication technologies. Thus, these devices in the lowest level should also provide an interface for services and information that can be accessed by any other node of the system, to incorporate them into the control and monitor processes.

For this, the standard IEEE 1451 describes the concept of a smart transducer and its capabilities. A smart transducer is defined as a physical device that provides more functionalities, beyond those needed for acquiring a sensed quantity or controlling a specific mechanism. Those functionalities simplify the interconnection and integration of the transducers in a new or existing environment. Furthermore, an IEEE 1451 smart transducer affords capabilities for self-identification, self-description, self-diagnosis, self-calibration, location-awareness, time-awareness, data processing, reasoning, data fusion, alert notification, and communication protocols [3]. Although these characteristics are necessary to provide plug-and-play features to smart transducers, this standard on its own is not sufficient to support the automatic recognition of these IEEE 1451 compliant transducers (physical side of the system) on a CPPS application.

On the cyber side of the system, the smart transducers will have a digital representation and will be integrated into monitoring or control processes. The set of these processes forms an application that can be designed following the standard IEC 61499. This standard defines a distributed and service-oriented architecture, based on the construction of applications using event-triggered function blocks (FB). However, despite the fact the standard allows one to have a function block representation of a smart transducer, the system is still not capable of discovering new devices and self-reconfigure the application.

Essentially, although these two standards are solid and sustainable on their own, it is necessary to enable interoperability between them and add support for plug and play and system reconfigurability for the integration of new physical devices. Therefore, the main purpose of this work was to develop an edge device with plug-and-play capabilities that integrated the two standards, creating a platform that established a connection between a smart transducer compliant with IEEE 1451 and an application designed according to the IEC 61499 standard.

### Research Questions

The main goals of this work were the development of an edge device that interoperated the IEEE 1451 and IEC 61499 standards and provided a solution to integrate smart sensors automatically in industrial applications in a plug-and-play approach. This led to the following research questions (RQs):

- **RQ1:** How can the IEEE 1451 and IEC 61499 standards be integrated to embed smart transducers in industrial applications?
- **RQ2:** What plug-and-play level can be achieved in the industrial context?
- **RQ3:** What advantages can be drawn from the automatic integration of smart transducers compliant with IEEE 1451?

## 2. Background and Related Work

### 2.1. IEEE 1451—Smart Transducers

A smart transducer is a device that aggregates an analog or digital sensor or an actuator, a signal condition and data conversion module, a processing unit, and a network communication interface [3]. With the outgrowth and demand for integrating new technologies, namely smart transducers, verified in the industry, the IEEE 1451 family of standards [4,5,6,7,8,9,10,11] was defined to standardize the connectivity of transducers to industrial networks, describing the smart transducer’s architecture, interfaces, services, and communication between it and the network [12].

**Figure 1:**
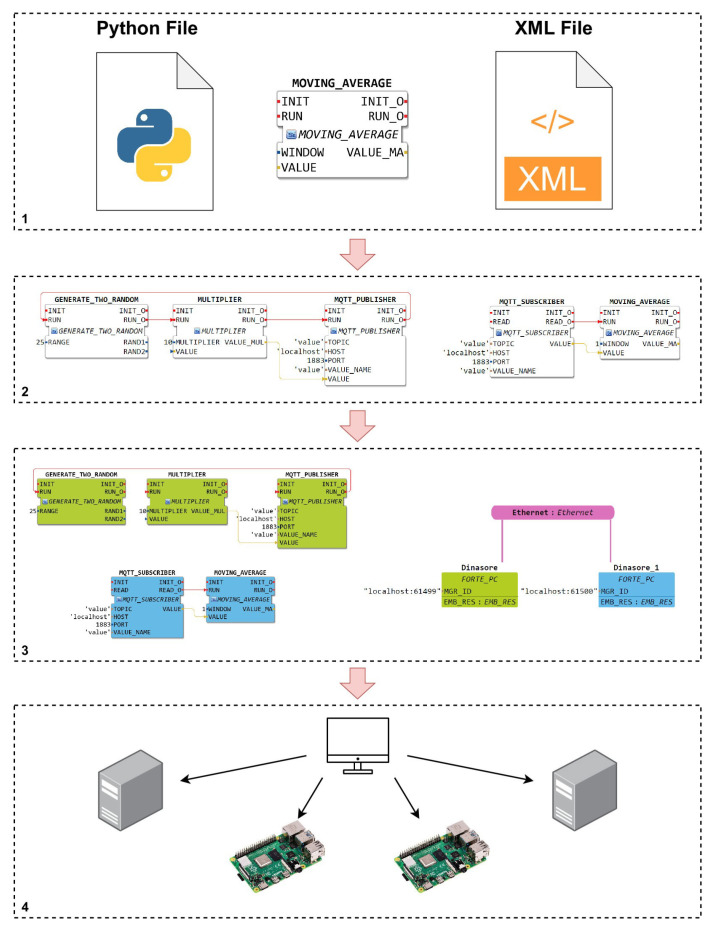
4diac IDE and DINASORE—Development Process.

Therefore, the IEEE 1451 standards extend the initial definition of a smart transducer, adding new capabilities, such as the ability for self-description using the transducer electronic data sheet (TEDS) concept. This family of standards divides the system into two components—the network capable application processor (NCAP) and the transducer interface module (TIM)—and defines the interface between the TIM and the NCAP as the transducer independent interface (TII) and between the NCAP and the network as the network interface (NI). The complete smart transducer model is defined in Figure 1 of the IEEE 1451.0 standard [4].

The transducer electronic data sheet is a standardized specification of the manufacturing information, including the manufacturer information and the information typically presented in the transducer datasheet, such as measurement range, accuracy, and calibration data. Thus, when a smart transducer is detected in a network, the system can request its TEDS to know the transducer information and the services available.

Redefining the concept of a smart transducer and providing a set of standardized interfaces for connecting these transducers to a system’s network, the IEEE 1451 standard helps to achieve sensors and actuators’ plug-and-play capabilities and interoperability [3]. However, automatic plug and play is only possible at the transducer level and still depends on the system’s ability to recognize transducers and put them available to the users and developers [13].

### 2.2. IEC 61499—Distributed Industrial Systems

The IEC 61499 standard [14] defines an architecture for distributed systems development. This architecture is based on function blocks (adapted from the older IEC 61131-3 standard to a new event-triggered perspective). Each function block (FB) represents a self-contained software unit with its own variables and algorithms, that is connected to other FBs through its external interface (input and output), which includes events and data associated with those events. Once each FB can run on different devices, the system’s intelligence is decentralized and embedded into different nodes distributed across the network [15,16].

**Figure 2:**
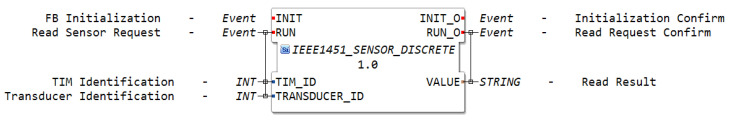
Smart Function Block—IEEE 1451 Sensor (Discrete).

The development of an application compliant with IEC 61499 consists of the definition of those function blocks and a posterior interconnection to form a function block network (in order to create a functional and logical structure). Then, software is deployed to the hardware components available on the system. As shown in Figure 2 from article [16], not only can an application be distributed across multiple devices, but a device can also support multiple applications.

In this way, the IEC 61499 standard is defined as the primary choice to create distributed industrial automation solutions [15] due to the portability, interoperability, and configurability offered by a function-block-based application [17]. However, the FB instances’ management still needs further developments to achieve and support plug-and-play capabilities and the integration with other industry standards. Even though the standard supports reconfigurability between the existent FBs of an application, the association of a new device is not immediate and manual work is needed to create a new function block. For instance, the integration of a new sensor in an application involves the manual development of a new function block that encapsulates the driver of that sensor and the manual addition of the FB to the application editor (e.g., 4diac IDE).

#### 4diac IDE and DINASORE Framework

As described in the previous section, the standard IEC 61499 defines a solution for developing applications for distributed industrial systems. Therefore, it implicitly defines the use of an integrated development environment (IDE) to build the application and a runtime environment (RTE) to run the application.

The 4diac IDE (4diac IDE and 4diac FORTE are available online at https://www.eclipse.org/4diac (accessed on 31 July 2022)) is an example of an IDE compliant with the IEC 61499 standard. This software environment is based on the Eclipse framework and focuses on the development of applications that can be deployed to different devices available in the system. However, the 4diac IDE does not natively support the automatic appearance and configuration of function blocks in the editor.

In other respects, DINASORE [18] is an example of a runtime environment compliant with IEC 61499 and compatible with the 4diac IDE. DINASORE is an open-source project (DINASORE project is available online at https://github.com/DIGI2-FEUP/dinasore (accessed on 31 July 2022)) developed by the DIGI2 Laboratory based on 4diac FORTE but written in the Python language. Besides running IEC 61499 applications built with function blocks, once it is written in Python, DINASORE supports the latest developments in artificial intelligence that has seen large developments in that language. Concerning its specific implementation, DINASORE uses a producer–consumer pattern to exchange events and data (interfaces of a function block) through the function block network, where each FB is running in a different thread.

Thus, the complete development process of IEC 61499 applications using the 4diac IDE and DINASORE framework can be summarized as follows and as illustrated by Figure 1:

1. Build new function blocks, writing the executable code in a Python file, and defining its interfaces in an XML file;
2. Draw the application (function block network), dragging and dropping function blocks and interconnecting them in the 4diac IDE editor;
3. Map each function block to the device (running DINASORE) that should execute it;
4. Deploy the solution to the respective devices.

Summing up, the 4diac IDE and DINASORE framework can and is used in the development of the edge device as the IDE and RTE, to be compliant with IEC 61499 standard. The other components of the edge device are responsible for integrating the recognition and use of smart sensors compliant with the IEEE 1451 standard.

### 2.3. IEEE 1451 and IEC 61499 Interoperability

Despite the fact that the IEC 61499 standard provides important functionalities on portability, interoperability, and configurability to a distributed system, it does not natively support the integration of IEEE 1451 compliant smart transducers [19].

In [13], a solution was proposed to enable the interoperability between both standards and a runtime platform to test the proposed architecture, in order to take advantage of IEC 61499 features to perform processing and system integration at a low level (transducer level), facilitating smoother plug-and-play actions, as defined in the IEEE 1451 standard. Since IEC 61499 does not support TEDSs and TIMs, the interconnection was done throughout the NCAP. Therefore, IEC 61499 devices and NCAPs from IEEE 1451 transducers were connected to the same network and exchange messages via a client/server or publisher/consumer protocol (both supported by the two standards).

As a result, the application could be deployed on devices (IEC 61499) and NCAPs (IEEE 1451). Furthermore, with this architecture, reconfigurability could be achieved either by adding or removing NCAPs from the network or sensors/actuators from the TIM, expanding plug-and-play capabilities and (re)configurability to the transducer’s level.

The platform used in [13] to test the proposed solution was composed of a Raspberry Pi with 4DIAC FORTE (RPi FORTE), representing an IEC 61499 device, and a Raspberry Pi with an NCAP program developed in Python that communicated via a UART wired connection with a TIM (Texas Instruments microcontroller), representing an IEEE 1451 device. The RPi FORTE and the NCAP communicated with each other by MQTT, throughout Mosquitto (an MQTT broker).

Moreover, the authors in [13] did not specify any other application besides the use of the NCAP as a communication bridge between the network and the TIM and other communication protocols besides MQTT in the network interface and serial in the transducer independent interface.

In [20], a relation was established between the communication layer that implements the interoperability among the standards IEEE 1451 and IEC 61499 (as defined in [13]) and the Industrial Internet Reference Architecture. It also presented the results of the use of HTTP and MQTT communication between IEC 61499 devices and IEEE 1451 NCAPs available in local and external networks. These communication protocols corresponded to the application layer of the OSI model, enabling the syntactical level of interoperability of both standards.

Therefore, the importance of achieving interoperability between the standards IEEE 1451 and IEC 61499 is recognized. However, running both standards’ names as keywords in academic search engines (e.g., Google Scholar, IEEE Xplore, and MDPI), we could not identify any other relevant works on the implementation of that interoperability besides those presented in this section ([13,20]). In this way, new developments are important to expand the functionalities of NCAPs and their communication protocols to the network and to TIMs, to support the integration with other standards. Furthermore, the native integration between both standards should also be studied, i.e., not only the use of common gateways and communication protocols to combine both standards.

### 2.4. Plug and Play

The industry transformation in terms of flexibility and adaptivity of the production environment, due to the continuous change of products, technologies, and resources, evidences the need to add plug-and-play (PnP) abilities to a CPPS, particularly to its shop floor components (e.g., sensors, actuators, and controllers). These components can be seen as “Smart Components”, with self-description [21,22] and self-discovery [12] capabilities.

According to [23], the system should be able to recognize these smart components and their self-description, connect them to the specific control or monitor loop, and start or continue the operation without change in the rest of the production system. Therefore, plug and play can be defined as the automatic recognition of a new or modified component in a production system and its correct integration into the ongoing processes without manual intervention, downtime, and changes in the implementation of the remaining production system [23,24].

The recognition and integration of unknown devices into a CPPS in a plug-and-play approach require, according to [25], five steps: (1) physical connection, (2) discovery, (3) basic communication, (4) capability assessment, and (5) configuration. Taking the IEC 61499 architecture as an example, after the automatic configuration and integration of a device, one needs to represent it in a function block, in order to make its services and information available to a distributed application. Then, a sixth step can be stated in addition to the presented steps, which is the integration of the device into existing applications.

Different proposals for the implementation of a CPPS are reviewed in [26] and service-oriented architectures (SOA) are identified as the key to enabling flexibility and collaboration with plug-and-play solutions. Furthermore, in a plug-and-play perspective, once the components provide well-defined interfaces to access their services, the system is able to self-configure and self-recover. The question is how to describe the interfaces and make them available to the system. For this, the standard IEC 61499 defines a service-oriented architecture based on function blocks, that can be used in the development of distributed industrial applications.

## 3. Architecture

The integration of smart transducers in industrial applications based on the IEEE 1451 and IEC 61499 standards presented in this work occurs on two different levels. The first is a semantic and syntactic level related to the definition of function blocks that represent a smart transducer (smart function blocks). The second is a code integration level, developing an edge device that implements the concept of NCAP from IEEE 1451 and executes the smart function blocks in DINASORE.

### 3.1. Smart Function Block Definition

The interoperability between the IEEE 1451 and IEC 61499 standards presented by this solution is based on the representation of a smart transducer compliant with the IEEE 1451 standard by a function block defined in the IEC 61499 standard. Once these function blocks are oriented to run on DINASORE, their events are defined accordingly to the DINASORE architecture [18].

The IEEE 1451 standard defines 3 smart transducer types: (1) sensor, (2) event sensor, and (3) actuator. One or more function blocks can be formally defined, which represent each type of smart transducer. These function blocks can be named smart function blocks.

**Figure 3:**
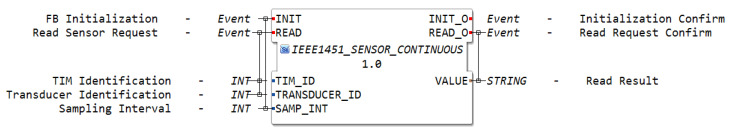
Smart Function Block—IEEE 1451 Sensor (Continuous).

A sensor is a type of transducer that measures some physical entity and returns a digital representation of the measured value. To this type of transducer, two different smart function blocks were defined. One to read the sensor value discretely (Figure 2) and the other to read it continuously (Figure 3).

**Figure 4:**
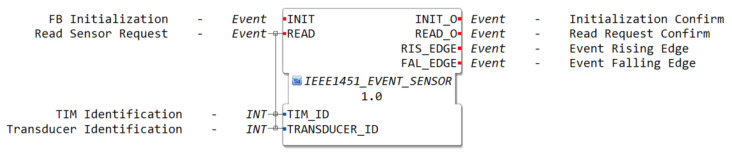
Smart Function Block—IEEE 1451 Event Sensor.

An event sensor is a type of transducer that, instead of measuring and returning the value of a physical entity, detects when a change of state has occurred, i.e., it can be configured to detect threshold crossings, bit patterns, or define hysteresis. The corresponding function block is presented in Figure 4, which triggers an output event in the case of a rising edge or falling edge in its value.

**Figure 5:**
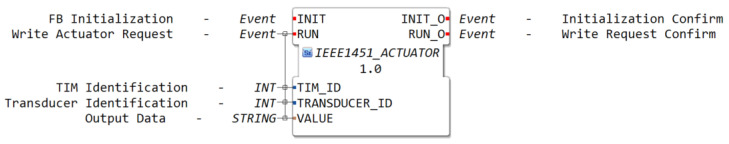
Smart Function Block—IEEE 1451 Actuator.

An actuator is a type of transducer that changes the value of a physical entity, according to the data it receives. The representation of an actuator in a function block is presented in Figure 5.

These four smart function blocks that map the IEEE 1451 standard transducers into IEC 61499 standard function blocks are the basis of the integration of the NCAP with the 4diac IDE and DINASORE.

### 3.2. Edge Device Architecture

The edge device solution proposed by this paper arises from the combination and interoperability of the IEEE 1451 and IEC 61499 standards, particularly the NCAP definition in the IEEE 1451.0 standard and the 4diac IDE and DINASORE framework based on the IEC 61499 standard.

**Figure 6:**
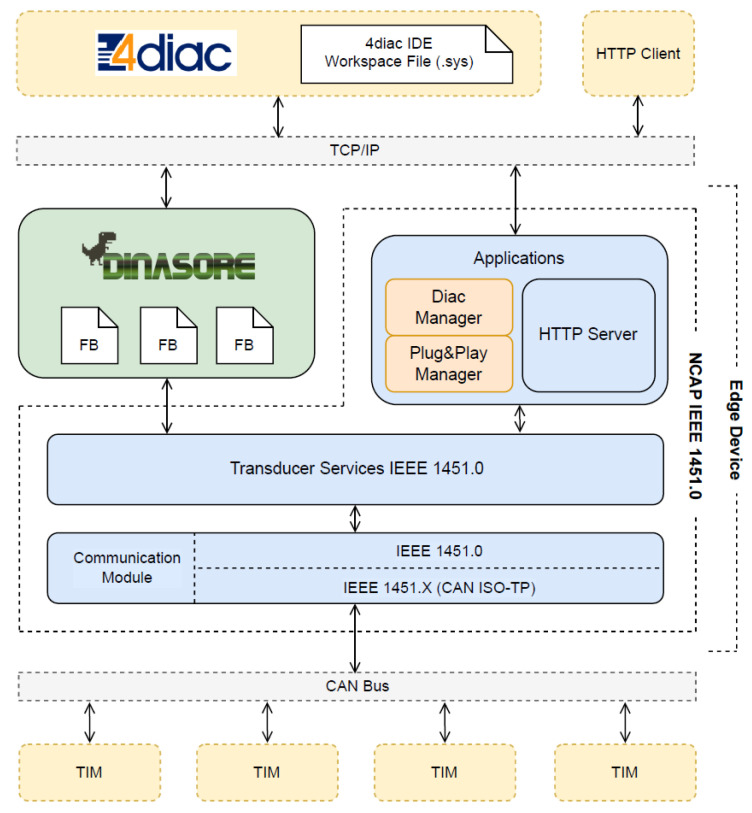
Edge Device Architecture.

The high-level architecture of the edge device is presented in Figure 6. This diagram shows the main components of the system and how they interact with each other.

In this architecture, there is a symbiotic relationship between DINASORE and NCAP. On the one hand, DINASORE acts as a runtime environment of IEC 61499 control applications that can be distributed by multiple nodes in a cyberphysical system. On the other hand, the NCAP is responsible for automatically detecting and making available the sensors and actuators existing in the system. The symbiosis occurs from the moment when DINASORE accesses those sensors and actuators inside a smart function block and integrates them into an application.

From the point of view of the NCAP, its structure followed the IEEE 1451 reference model. The transducer services interface, the NCAP IEEE 1451.0 services, the module communications interface, and the NCAP IEEE 1451.0 communication module were developed accordingly to the IEEE 1451.0 standard. The IEEE 1451.X communication module was implemented to support the ISO-TP standard over the CAN bus protocol as the physical communication protocol with TIMs.

As the NCAP was intended to work in a stand-alone version, i.e., decoupled from DINASORE, it also implemented an application level. The NCAP application level consisted of two applications: (1) an HTTP server that handled requests from external users’ applications and (2) a “Plug & Play (PnP) Manager” that monitored the connection of new transducers and notified any registered observer. The HTTP server was defined by the standard, whereas the PnP manager was defined entirely by the authors. A “Diac Manager” is an example of an observer that needs to know when a new transducer is added to the system. When that happens, the “Diac Manager” changes the workspace file of the 4diac IDE, and the smart function block automatically appears in the IDE.

From the point of view of the DINASORE, the development process involved the implementation (Python and XML) of the smart function blocks that represented a sensor or actuator and finding a strategy to pass the NCAP object into the FB execution function. Once it was possible to access the NCAP object inside a function block, it could access directly the NCAP services through its transducer service interface.

In this way, besides the automatic configuration of the sensors as defined in standard IEEE 1451, the edge device allows for a complete plug and play from the connection of a sensor until the integration of that sensor in the application (the sixth plug-and-play step introduced before in Section 2.4).

## 4. Validation Scenario, Performance Tests, and Results

This section presents a validation scenario for the edge device. This scenario was based on the FactoRIS project, whose development highlighted the importance of integrating smart sensors in a plug-and-play approach to reduce the development time. In the context of this validation scenario, performance tests were realized to verify the limits of the edge device, concerning the registration and discovery time and read/sampling time.

### 4.1. FactoRIS Validation Scenario

The validation scenario for the developed edge device was the FESTO Modular Production System used to create a learning factory under the FactoRIS project, hosted by the DIGI2 Laboratory and supported by EIT-Manufacturing.

The FactoRIS project (“Learning Factories for Digital Transformation of SMEs”) intends to contribute to the digital transformation of SMEs by creating learning factories where new technological advances can be used to solve digitalization challenges [27]. One of the solutions developed in the FactoRIS project was a condition monitoring system to monitor a production line composed of FESTO stations (more information on the FESTO Modular Production System is available at https://www.festo.com (accessed on 31 July 2022)) [28].

Part of the actuating system of the FESTO stations is composed of pneumatic actuators, whose components degrade over time. To cope with this degradation, air flow and pressure analog sensors were installed in the pneumatic system to monitor and detect any air leak or other abnormal behaviors (as suggested in Figure 2a) from article [28]).

The integration of those sensors in the application was done manually, using an ADC for Raspberry Pi, developing custom function blocks to read and convert the analog values, and manually adding the function blocks in the 4diac IDE.

With the edge device proposed and developed in this work, the integration of those sensors in the condition monitoring application can be done faster and with much less human effort. To test the edge device, we considered the following situation:

- Two flow sensors and two pressure sensors needed to be connected to the application. Thus, each one of the sensors was connected to a different TIM, which resulted in the connection of four TIMs in the same CAN bus.

**Figure 7:**
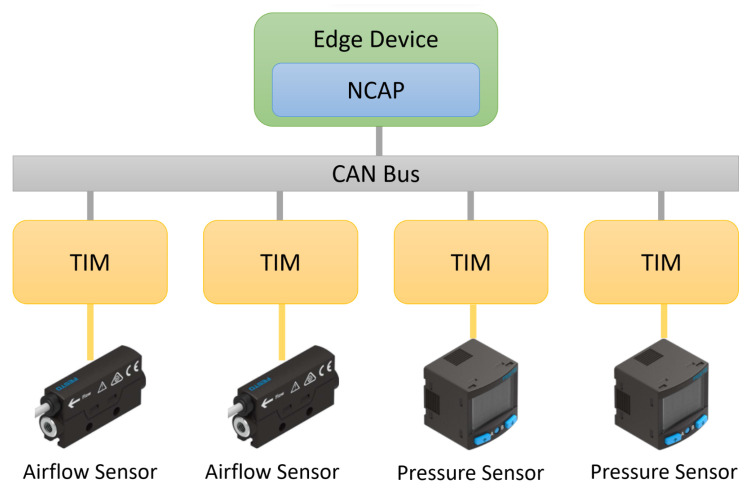
Test Scenario.

**Figure 8:**
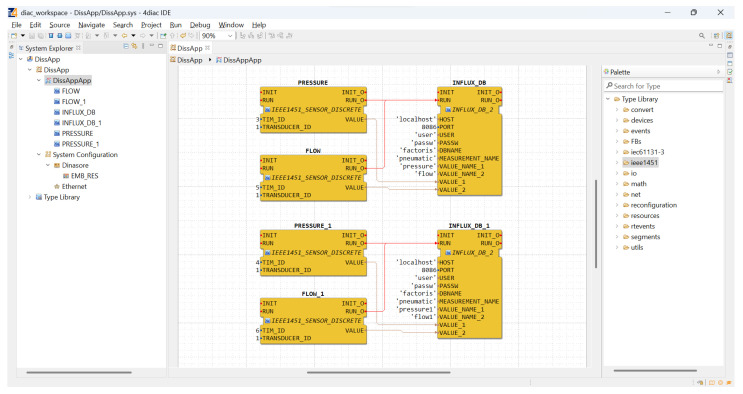
4diac IDE application—Test Scenario.

This scenario is illustrated in Figure 7:

When a new transducer (or TIM) was added to the network, the NCAP read its TEDS and, based on the type of transducer, added a new function block to the application editor (4diac IDE). Internally, when the function block was executed, it accessed the NCAP services to read/actuate the respective transducer. In this scenario, the flow and pressure blocks in Figure 7 were integrated in a plug-and-play approach, being automatically added to the IDE and becoming readily available to be used by the application (represented in Figure 8).

### 4.2. Performance Tests

The performance tests consisted of determining how a system performed in terms of speed, responsiveness, and stability under different workloads. In this work, we developed an NCAP that, integrated with DINASORE, constituted an edge device to integrated smart transducers in industrial applications.

To test the plug-and-play feature, we performed tests on the registration and discovery of TIMs available on the network. Furthermore, once a TIM was connected to the NCAP, their interaction was based on the exchange of commands, then we also tested the sending of a Read Data command. These performance tests could be used to compare different communication protocols implemented between the NCAP and TIMs. In this particular case, it was tested with CAN ISO-TP.

The performance tests were based on measuring and analyzing the total time of the action with multiple TIMs in parallel, i.e., with more and less workload in the system. Due to hardware stock limitations on the TIM side, the tests were only performed with up to four TIMs. Further tests should be performed in order to make conclusions on a regression analysis and tendency functions. Each measured time interval presented in the following sections is in fact an average of 20 samples.

#### 4.2.1. Registration Time Tests

When a TIM is turned on, it needs to register itself in the NCAP. The registration process follows the following steps:

1. The TIM sends a Register command to the NCAP. The NCAP registers the TIM and responds with the new destId (CAN address).
2. The NCAP sends a Read TEDS command to get MetaTEDS;
3. If the MaxChan (number of implemented TransducerChannels) field of MetaTEDS is greater than zero, the NCAP sends Read TEDS commands to get the TransducerChannelTEDS of each transducer channel from that TIM. The NCAP registers each transducer channel with the associated TEDS;
4. The NCAP interprets the TransducerChannelTEDS of each transducer channel to get the physical units;
5. The NCAP adds the corresponding function block to the 4diac IDE workspace file.

This test intended to analyze the impact of the registration of multiple TIMs at the same time, i.e., when multiple TIMs were turned on simultaneously. The considered measurement interval started with the reception of the first CAN message and ended with the last closing of the workspace file. The results are presented in Table 1.

In this interval, depending on the length of each TEDS, a lot of CAN messages could be exchanged between the NCAP and the TIM, which led to a higher time interval.

As expected, there was a growth in the registration time when multiple TIMs were registered at the same time. Besides the large number of CAN messages that were exchanged in one registration process, when multiple TIMs were being registered, there were also CAN conflicts once multiple devices were trying to transmit a message.

However, even with registration times up to 2 s (with four TIMs), this value was very low compared to the time needed to manually develop, add and configure function blocks in the 4diac IDE.

#### 4.2.2. Discovery Time Tests

After the registration process, a TIM does not change its destId (CAN address) unless it is restarted. Therefore, when the NCAP is turned on, it needs to discover the TIMs available in the network that were already registered in an NCAP. The discovery process follows the following steps:

1. The NCAP sends a Discover command in broadcast to the network;
2. Each TIM that is already registered responds to the Discover command with its destId;
3. The NCAP registers internally a new TIM with the given destId;
4. The NCAP sends a Read TEDS command to get MetaTEDS for each TIM;
5. If the MaxChan (number of implemented TransducerChannels) field of MetaTEDS is greater than zero, the NCAP sends Read TEDS commands to get the TransducerChannelTEDS of each transducer channel from that TIM. The NCAP registers each transducer channel with the associated TEDS;
6. The NCAP interprets the TransducerChannelTEDS of each transducer channel to get the physical units;
7. The NCAP adds the corresponding function block to the 4diac IDE workspace file.

In this case, we measured the time to discover and register internally in the NCAP up to four TIMs available in the network. The considered measurement interval started with the start of the discovery process and ended with the last closing of the workspace file. The results are presented in Table 2.

Similarly to the results in the registration process, the presence of multiple TIMs had a high impact on the discovery process, which could also be explained by the large number of messages that needed to be sent at the same time. Comparatively to the values of the registration process, these values were higher once the process began in the NCAP and the time included a command message to the TIM, the processing of that message in the TIM, and the response to the NCAP.

These values (up to less than 2.5 s with four TIMs) were still a good time with respect to the plug-and-play functionality.

#### 4.2.3. Read Sensor Time Tests

After the registration of a TIM in the NCAP, its interaction is based on the exchange of commands defined by the IEEE 1451.0 standard. These commands are requested from the NCAP via its transducer services interface. During the normal operation of a TIM, the most common and essential command for a sensor-type transducer is the Read Data command. Therefore, tests were performed concerning this command.

The following tests measured the impact of the read of multiple sensors (transducer channels) in parallel, each one on a different TIM. These tests were divided into two measurements: (1) the time needed to execute a read service and (2) the maximum cycle time between consecutive reads. These two measurements were also measured in two different conditions: (1) with direct access to the transducer services, i.e., a thread was launched inside the NCAP’s main thread to each transducer channel, and (2) using DINASORE, i.e., using the complete edge device and the developed function blocks.

The results from the direct access to the transducer services inside the NCAP’s main thread are presented in Table 3 and Table 4.

Regardgin the read time, it is clear that a simultaneous operation led to a higher read time, explained by the same reason presented in the previous tests, i.e., because of the conflicts in the CAN transmission and more concurrent processing in multiple threads.

The read cycle time corresponded to the maximum sampling time and was close to the read time, once the next read operation was immediately started. Its inverse gave the maximum samples that could be read in one second, which with four TIMs was only approximately five samples per second. This value was low with respect to real-time applications, but could be sufficient for applications with few time restrictions.

It should be noted that in a read operation, the returned information is in fact a data set, i.e., reading a data set can result in one or more measuring values. Therefore, the real measuring rate should be obtained by multiplying the maximum number of samples per second by the number of values present in a data set (defined in TransducerChannelTEDS). However, the transmission of larger data sets may also impact the read and read cycle times.

Once the NCAP was integrated into a wider edge device that used DINASORE as a runtime environment, the behavior of the read operation using the complete edge device was also tested. The results are presented in Table 5 and Table 6.

With the use of DINASORE, the read time increased compared to the read time obtained with a direct access to the transducer services. With the use of DINASORE, more threads were running at the same time, which together with the NCAP threads and CAN communication, increased the read time up to approximately 250 ms with four TIMs.

Regarding the read cycle time, in contrast to the previous case, its value was not similar to the read time. This could be explained by the event trigger mechanism that was executed internally by DINASORE, which was slower than the simple while cycle used in the direct access to the transducer services. Thereby, the maximum number of samples obtained was 3.6 in the case of reading simultaneously the values from four TIMs.

As soon as a TIM is available that implements the referred data sets and provides them in read data operations, additional performance tests must be done to measure the impact of transmitting a larger data set in the CAN network and to analyze the respective cost-benefit ratio.

#### 4.2.4. Tests Summary

The validation scenario provided a real-case test bed where it was necessary to incorporate airflow and pressure sensors into a pneumatic actuation system and connect them automatically (plug and play) to a condition-monitoring application.

Regarding the concrete tests performed, it was concluded that the registration and discovery time (even for the connection of four sensors) was enough to favor a plug-and-play solution, reducing significantly the integration time of these sensors in the application.

In contrast, the read cycle time that could be obtained with this implementation was not very low, which yielded a maximum of 4.9 samples/s (without DINASORE) and 3.6 samples/s (with DINASORE), when considering the simultaneous read of four TIMs. However, these values were presumably overcome with the reading of data sets instead of single values, also defined in the IEEE 1451.0 standard.

## 5. Conclusions

In a cyberphysical production system, the portability and reconfigurability of the control and monitoring application are mandatory to achieve plug-and-play capabilities that support the integration of new sensors and actuators in a quick and simple way, without the need for engineering intervention to reconfigure the system. The developed solution integrated the IEEE 1451 and IEC 61499 standards and allowed the automatic recognition and configuration of smart transducers in distributed industrial applications.

Based on the content exposed, the following answers to the research questions can be stated:

**RQ1:** The interoperability between the IEEE 1451 and IEC 61499 standards in the development of industrial applications can be carried out from two different perspectives. In the related work, there were solutions that implemented a common communication protocol between devices compliant with each standard. In contrast, the solution presented in this work integrated both standards at a syntax level, i.e., representing the smart transducers compliant with the IEEE 1451 standard with a function block from the IEC 61499 standard.

**RQ2:** The integration of sensors and actuators in industrial applications with a plug-and-play approach must be classified by taking into account the level of integration that can be achieved. The automatic representation of the function blocks in the development platform and its posterior connection to the existing function block network constitutes a complete plug-and-play solution from the connection to the network until its integration in control and monitor applications.

**RQ3:** A smart transducer compliant with IEEE 1451, besides providing more advanced services beyond simple measurements, has self-description capabilities (due to the implementation of TEDS). In this way, it contributes to the creation of plug-and-play solutions, reducing significantly the development time of industrial systems.

In this way, from the point of view of the physical entities (sensors and actuators) of a CPPS, if they implement the IEEE 1451 standard, then it is possible to integrate them into the application since the standard guarantees compatibility. If the device is not compliant with IEEE 1451, but its manufacturer provides its datasheet, an abstraction from the standard can be done to access that sensor via its communication protocol (ex: SPI, I2C) and integrate it into the application, as it is in a normal TIM.

Summing up, the solution presented in this work allowed for the integration of self-described smart transducers in industrial applications in a plug-and-play approach, i.e., without human effort, in the development of custom function blocks and their respective configuration in the application designer. Once integrated directly within a development framework (4diac IDE and DINASORE) that is used nowadays in the development of cyberphysical systems, this solution constitutes a technological advance in this field and can actually be adopted in that development.

## 6. Future Work

Further improvements on the NCAP module of the edge device can be done to implement nonblocking operations at the transducer services level and data streaming from the TIMs to the NCAP, to support more communication layers for different physical protocols, in particular, those defined by the standards of the IEEE 1451 family, and to detect the disconnection of a TIM from the network.

At the IEC 61499 standard level, i.e., associated with the integration with the 4diac IDE and DINASORE framework, future work can be done on the automatic connection of the function blocks to the function block network available in the 4diac IDE. At this moment, only the function block is added to the 4diac IDE editor, thus the connections must be done manually. If we take into account the localization of the transducer (given by the geographic location TEDS) or the type of sensor/actuator (given by the TransducerChannelTEDS), and there is a block in the FB pipeline that asks for that specific function block, the connection can be done automatically.

## Figures and Tables

**Table 1:** Registration Test—Total Times, *n* TIMs.

# TIMs	Registration Time ($\bar{\Delta t}$, ms)
1	714.315
2	858.835
3	1237.022
4	2190.061

**Table 2:** Discovery Test—Total Times, *n* TIM.

# TIMs	Discovery Time ($\bar{\Delta t}$, ms)
1	852.134
2	1197.068
3	1777.288
4	2385.188

**Table 3:** Read Sensor Tests—Total Times, direct access to TS.

# TIMs	Read Time ($\bar{\Delta t}$, ms)	Average ($\bar{\Delta t}$, ms)
1	78.089	78.089
2	113.195	113.329
	113.462	
3	161.224	161.529
	161.789	
	161.575	
4	201.695	203.051
	203.801	
	203.600	
	203.107	

**Table 4:** Read Sensor Tests—Read Cycle Times, direct access to TS.

# TIMs	Read Cycle Time ($\bar{\Delta t}$, ms)	Average ($\bar{\Delta t}$, ms)	Max Samples/s
1	78.096	78.096	12.805
2	113.202	113.335	8.823
	113.469		
3	161.231	161.536	6.191
	161.796		
	161.581		
4	201.702	203.057	4.925
	203.807		
	203.607		
	203.114		

**Table 5:** Read Sensor Tests—Total Times, with DINASORE.

# TIMs	Read Time ($\bar{\Delta t}$, ms)	Average ($\bar{\Delta t}$, ms)
1	130.559	130.559
2	153.209	153.585
	153.961	
3	178.312	176.240
	180.360	
	170.047	
4	257.120	246.113
	253.741	
	235.548	
	238.044	

**Table 6:** Read Sensor Tests—Read Cycle Times, with DINASORE.

# TIMs	Read Cycle Time ($\bar{\Delta t}$, ms)	Average ($\bar{\Delta t}$, ms)	Max Samples/s
1	158.704	158.704	6.301
2	172.856	172.981	5.781
	173.107		
3	220.609	211.636	4.735
	215.749		
	198.550		
4	292.580	274.231	3.662
	280.840		
	246.274		
	277.228		

## Data Availability

Not applicable.
